# Supplementary material for: Identifying crossovers in a cattle pangenome containing haplotype-resolved assemblies from half-siblings
Source: BMC Genomics. 2026 Aug 7;27:914. doi: 10.1186/s12864-026-13232-6 (PMC13732852; doi:10.1186/s12864-026-13232-6)
Supplement: Supplementary file 1 [file 12864_2026_13232_MOESM1_ESM.zip › Supplementary_Figure_2.pdf]

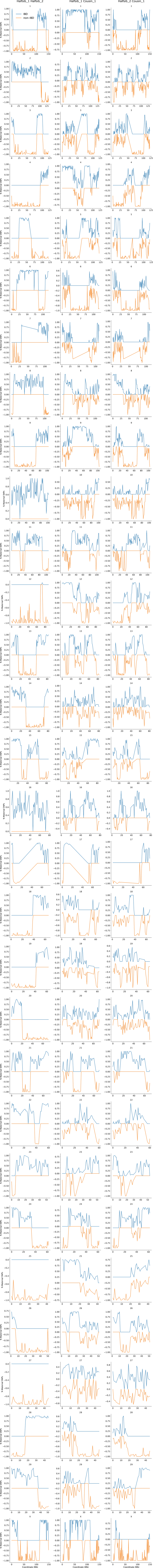
Halfsib_1 Halfsib_2
Halfsib_1 Cousin_1
Halfsib_2 Cousin_1
IBD
non-IBD
% Maternal SNPs
Coordinate (Mb)
